# Genetic diversity of HIV in Yunnan, China: the role of second-generation recombination involving circulating and unique recombinant forms

**DOI:** 10.1186/s12985-025-02863-y

**Published:** 2025-07-14

**Authors:** Ying-Na Xie, Zheng-Xu Li, Ya-Ting Chen, Yong-Juan Li, Zhu-Qian Yang, Yuan Ren, Zi-Xuan Yang, Xin Chen

**Affiliations:** 1 Department of Pathogenic Biology, School of Basic Medical Sciences, Gannan Medical University, Ganzhou, 341000 China; 2 Department of HIV/AIDS and STD Prevention and Control, Baoshan Center for Disease Control and Prevention, Baoshan, 678000 China; 3 Baoshan People’s Hospital, Baoshan, 678000 China; 4 School of Basic Medical Sciences, Baoshan College of Traditional Chinese Medicine, Baoshan, 678000 China

**Keywords:** HIV, Genetic diversity, Recombination, Revolution, Molecular epidemiology

## Abstract

**Background:**

The genetic diversity of HIV is significantly influenced by second-generation recombination events involving circulating recombinant forms (CRFs) and unique recombinant forms (URFs), which are crucial for the virus’s evolution and dissemination. The China-Myanmar border region is recognized as a focal point for inter-subtype recombination of HIV, with recombinant strains predominating in this area.

**Methods:**

Near full-length HIV genomes were amplified from plasma samples of eight Burmese individuals newly diagnosed with HIV in Baoshan, China, from 2006 to 2020. Phylogenetic trees were constructed using maximum likelihood methods, and Bootscan analysis was conducted to identify recombination structures.

**Results:**

Among the eight sequences, one (YN33F28) clustered with subtype C, and one (YN9M24) with CRF08_BC. The remaining six sequences did not cluster with any known HIV subtypes, indicating they might represent novel recombinant strains. Bootscan analysis revealed that three sequences (YN36F38, YN35F22, and YN32M22) were likely formed through second-generation recombination involving known CRFs (CRF82_cpx and CRF86_BC) and a URF (KY406739). Additionally, three sequences (YN34F21, YN7F27, and YN8F28) were identified as newly formed URFs, resulting from complex recombination events between HIV subtypes B, C, and CRF01_AE.

**Conclusion:**

These results underscore the continuous evolution of HIV via recombination in the China-Myanmar border region. The identification of second-generation recombinants and newly formed URFs highlights the importance of continuous molecular surveillance to better understand HIV diversity and to inform strategies for prevention, treatment, and vaccine development.

**Supplementary Information:**

The online version contains supplementary material available at 10.1186/s12985-025-02863-y.

## Background

Acquired immunodeficiency syndrome (AIDS), caused by the human immunodeficiency virus (HIV), represents a significant global health challenge. By the end of 2023, there were approximately 39.9 million people living with HIV (PLHIV) worldwide [1]. Based on differences in gene sequences, HIV can be classified into HIV-1 and HIV-2^2^. HIV-1 encompasses group M, group N, group O, and group P [2]. Among these, group M viruses are the primary pathogens responsible for the global AIDS epidemic [3]. Group M can be further subdivided into subtypes such as A, B, C, D, F, G, H, J, K, and L, as well as unique recombinant forms (URFs) and circulating recombinant forms (CRFs) generated by recombination among subtypes [3, 4]. Inter-subtype recombination has substantially enhanced HIV’s genetic diversity, facilitating its rapid adaptation to selective pressures [5]. This has also posed greater challenges to the prevention, control, diagnosis, treatment, and vaccine development for AIDS.

The China-Myanmar border region is a hotspot for inter-subtype recombination of HIV, and recombinant strains have become the predominant strains in this region [6]. As of December 2024, more than 170 HIV CRFs have been identified globally, with over 20 discovered in the China-Myanmar border region [7]. Two of the main prevalent strains in China, CRF07_BC and CRF08_BC, were identified in this region in the early 1990s [8]. Since then, many CRFs and URFs, such as CRF82_cpx, CRF86_BC, and CRF160_0708, have been identified there [9–11]. Therefore, monitoring the molecular epidemiological characteristics of HIV in this region is crucial for understanding the latest trends in inter-subtype recombination of HIV.

Baoshan, a city in the western part of Yunnan Province, China, borders Kachin State in northern Myanmar. This study investigates the molecular epidemiological characteristics of HIV among newly reported Burmese PLHIV in Baoshan, China.

## Methods

### Participants

All Burmese individuals newly diagnosed with HIV in Baoshan between 2006 and 2020 were enrolled in this study. Relevant demographic data were collected, and five milliliter of whole blood was drawn from each participant using vacuum blood collection tubes. Plasma was separated from the whole blood by centrifugation and stored at -80 °C for subsequent viral RNA extraction.

### HIV near full-length genome amplification and sequencing

Viral RNA was extracted from 200 µL of plasma using the High Pure Viral RNA Kit (Roche Diagnostics Ltd., Mannheim, 11858882001). cDNA synthesis was performed using the PrimeScript II 1st Strand cDNA Synthesis Kit (TaKaRa Biomedical Technology Co., Ltd., Beijing, 6210 A). The HIV near full-length genome was amplified in three overlapping fragments using the TransTaq DNA Polymerase High Fidelity Kit (Beijing TransGen Biotech Co., Ltd., Beijing, AP131-13), as previously described [12]. Successfully amplified samples were identified by 1% agarose gel electrophoresis and sequenced using the ABI PRISM 377XL DNA sequencer (Applied BioSystems, California). For samples with successful sequencing of all three fragments, the HIV near full-length genome was assembled using SeqMan v7.1.0.

### Phylogenetic analysis

The top five sequences with the highest similarity to the query sequences were retrieved from the HIV sequence database using BLAST analysis [13] (Table S1). Additionally, HIV standard reference sequences of HIV subtypes were downloaded. All sequences—including the amplified sequences—were aligned, and a maximum likelihood tree was constructed in MEGA v7.0.26 using the Kimura 2-parameter model with 1000 bootstrap replicates. To validate the BLAST-based strain selection, a neighbor-joining tree was generated under the same parameters (Kimura 2-parameter model, 1000 replicates), incorporating reference sequences of all HIV subtypes and CRFs alongside the amplified sequences.

Bootscan analysis was conducted using SimPlot v3.5.1 to identify HIV inter-subtype recombination and characterize the recombination structure. The analysis was performed with 100 replicates, employing a 200-bp sliding window that advanced in 20-nt steps. The dataset comprised the amplified sequences along with their clustered similar sequences, using reference sequences of HIV subtypes B, C, F1, and CRF01_AE for comparison.

For strains sharing identical recombination breakpoints with known CRFs or URFs, maximum likelihood trees were constructed using the shared subregion fragments, under the same parameters (Kimura 2-parameter model, 1000 bootstrap replicates), alongside reference sequences of all HIV subtypes.

## Results

### Demographic information of the participants

Between 2006 and 2020, eight Burmese individuals were newly diagnosed with HIV in Baoshan, China. The participants included six females and two males, with ages ranging from twenty-one to forty-two years old in 2020, and ethnicities including Lisu, Dai, Han, and Jingpo (Table 1). Most participants were either uneducated or had only received primary education, and all were farmers. The majority were married, but three participants were unmarried. The primary route of HIV transmission was heterosexual contact, with only one male infected through needle sharing (Table 1).

**Table 1:** Characteristics of Burmese people living with HIV in baoshan, China

Participant	Birth year	Gender	Ethnicity	Education	Marital status	Occupation	Diagnose year	Transmission route	Sample year
YN7F27	1992	Female	Lisu	uneducated	Married	Farmer	2019	Heterosexual	2020
YN8F28	1991	Female	Dai	uneducated	Unmarried	Farmer	2019	Heterosexual	2020
YN9M24	1982	Male	Han	Junior high	Unmarried	Farmer	2007	Heterosexual	2020
YN32M22	1997	Male	Lisu	uneducated	Unmarried	Farmer	2020	Needle sharing	2020
YN33F28	1978	Female	Jingpo	uneducated	Married	Farmer	2006	Heterosexual	2020
YN34F21	1999	Female	Jingpo	Junior high	Married	Farmer	2020	Heterosexual	2020
YN35F22	1997	Female	Jingpo	Primary school	Married	Farmer	2020	Heterosexual	2020
YN36F38	1982	Female	Jingpo	Primary school	Married	Farmer	2020	Heterosexual	2020

### Prevalence of known subtypes of HIV strains

**Fig. 1:**
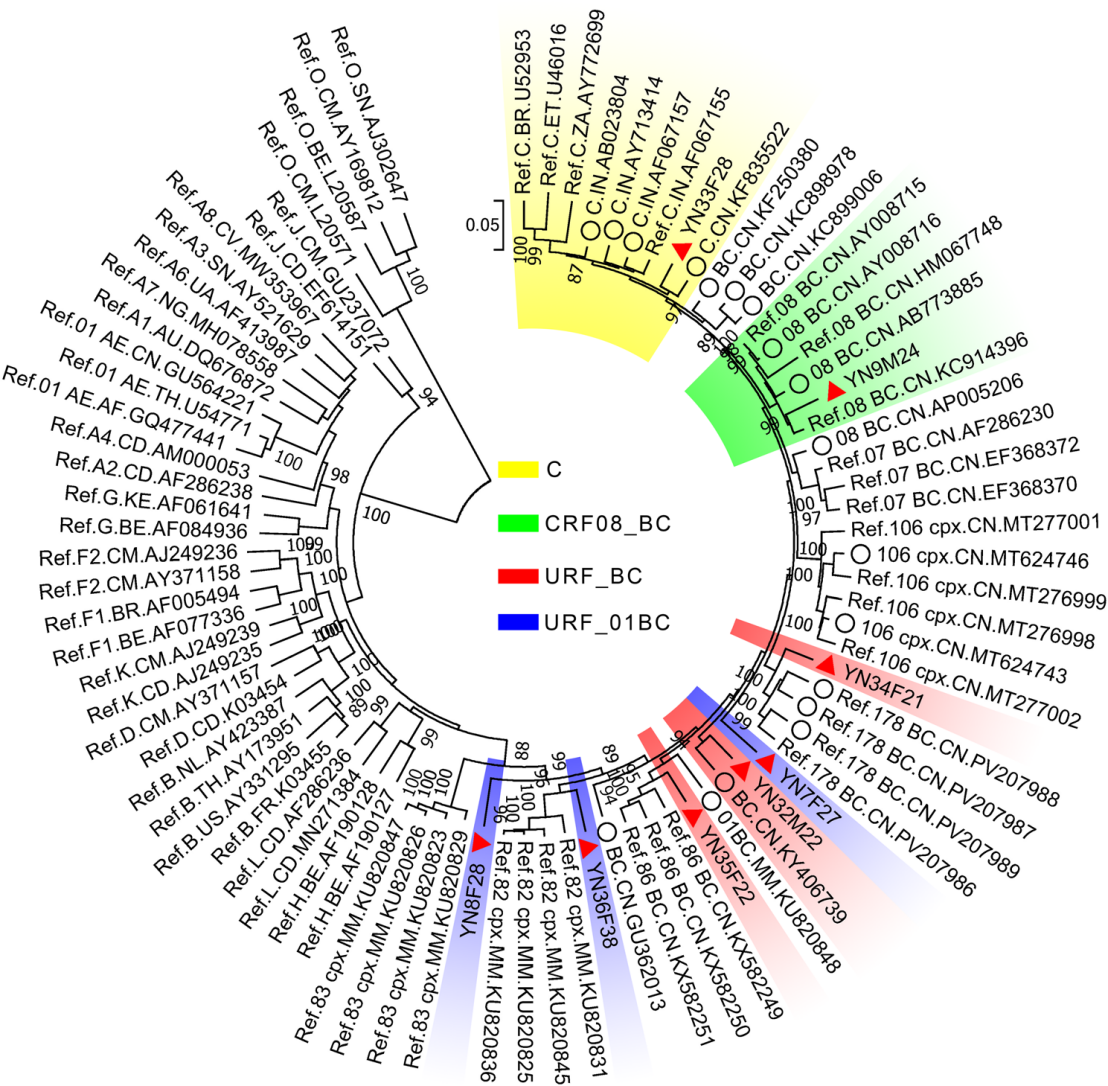
Maximum likelihood tree based on the near full-length genome of HIV. The red triangles represent the sequences amplified in this study, and the black circles represent the similar sequences downloaded from the HIV database. The sectorial shadings in different colors are used to distinguish different HIV subtypes

The maximum likelihood tree, constructed using near full-length genomes of HIV, showed that among the eight sequences amplified from Burmese PLHIV in Baoshan, only one sequence (YN33F28) clustered with the known subtype C, and one sequence (YN9M24) clustered with the known CRF08_BC, suggesting that they likely belonged to subtype C and CRF08_BC, respectively (Fig. 1). The remaining six sequences did not cluster with any known subtypes, indicating that they might have represented newly formed recombinant strains (Fig. 1). These findings were corroborated by a neighbor-joining tree incorporating reference sequences of all HIV subtypes and CRFs (Figure S1).

**Fig. 2:**
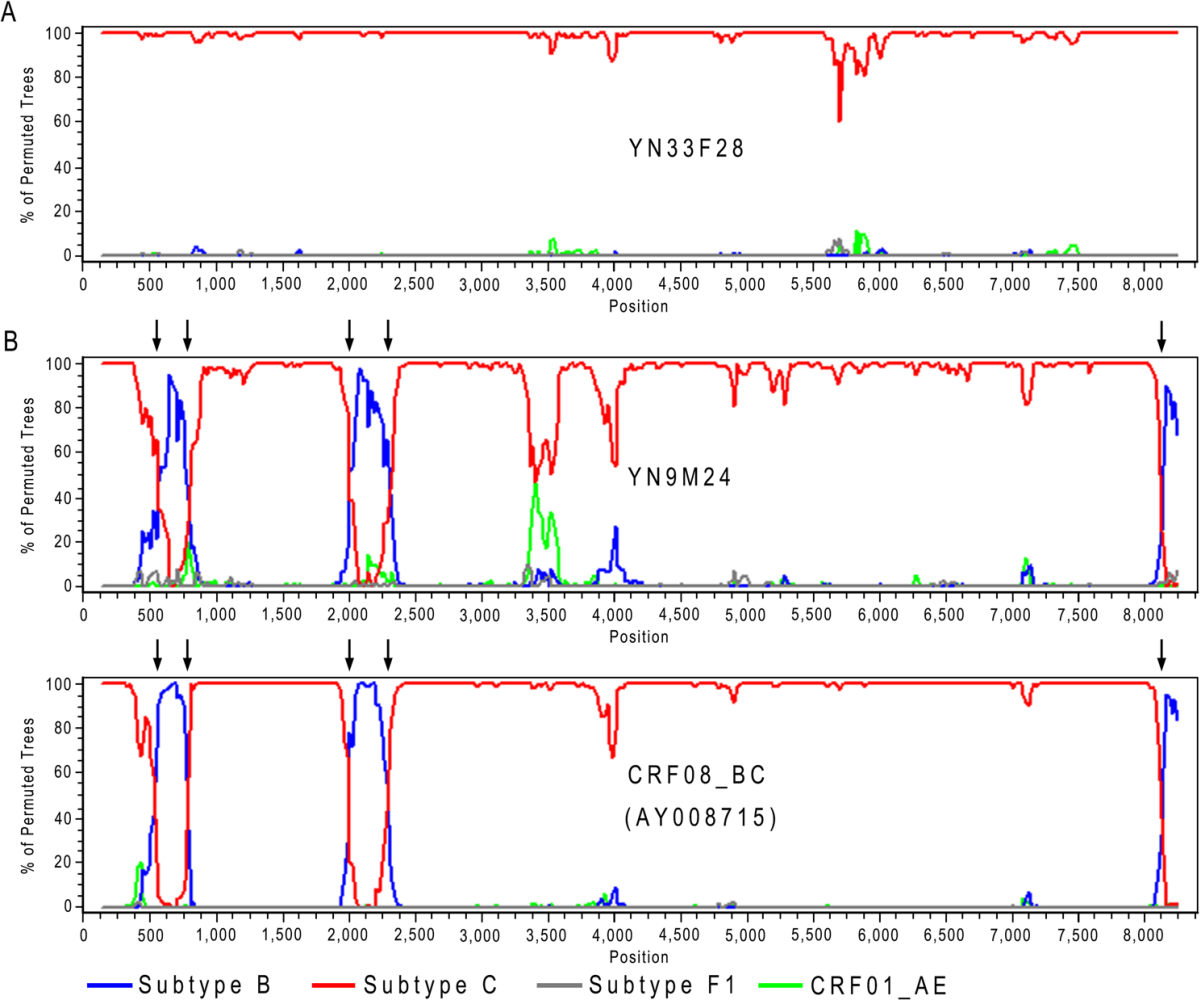
Bootscan plots of HIV sequences of known subtypes. (**A**) Subtype C; (**B**) CRF08_BC. In each plot, the lines in different colors represent the reference sequences of different subtypes, and the black arrows indicate the shared breakpoints among different HIV sequences

The Bootscan plot of sequence YN33F28 showed that it was most similar to HIV subtype C, with no recombination breakpoints, further confirming that it was indeed subtype C (Fig. 2A). The Bootscan plot of sequence YN9M24 demonstrated that its recombination structure was consistent with that of CRF08_BC, which was formed by recombination between subtype B and subtype C. It contained three subtype B fragments and three subtype C fragments, separated by five breakpoints (Fig. 2B). These results further confirmed that sequence YN9M24 belonged to CRF08_BC.

### Recombinant strains formed through second-generation recombination

The maximum likelihood tree revealed that sequences YN36F38, YN35F22, YN34F21, YN7F27, and YN32M22 clustered with known CRFs (CRF82_cpx, CRF86_BC, and CRF178_BC) and a URF (KY406739), respectively. However, these sequences were positioned closer to the root of the phylogenetic tree compared to their corresponding reference sequences, indicating that they were genetically closely related but not identical to the reference sequences. This suggested that they might have been recombinant strains formed through second-generation recombination involving these reference sequences (Fig. 1).

**Fig. 3:**
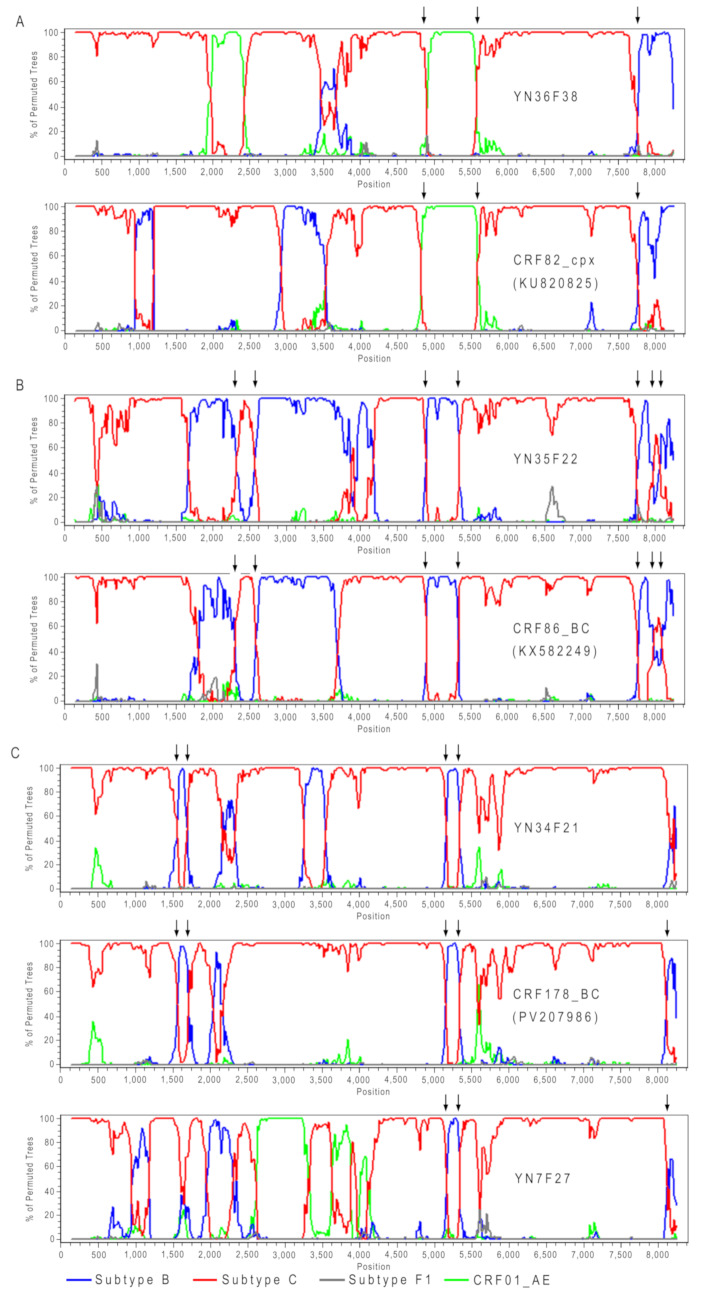
Bootscan plots of second-generation recombinant HIV sequences composed of CRFs. (**A**) CRF82_cpx; (**B**) CRF86_BC; (**C**) CRF178_BC. In each plot, the lines in different colors represent the reference sequences of different subtypes, and the black arrows indicate the shared breakpoints among different HIV sequences

**Fig. 4:**
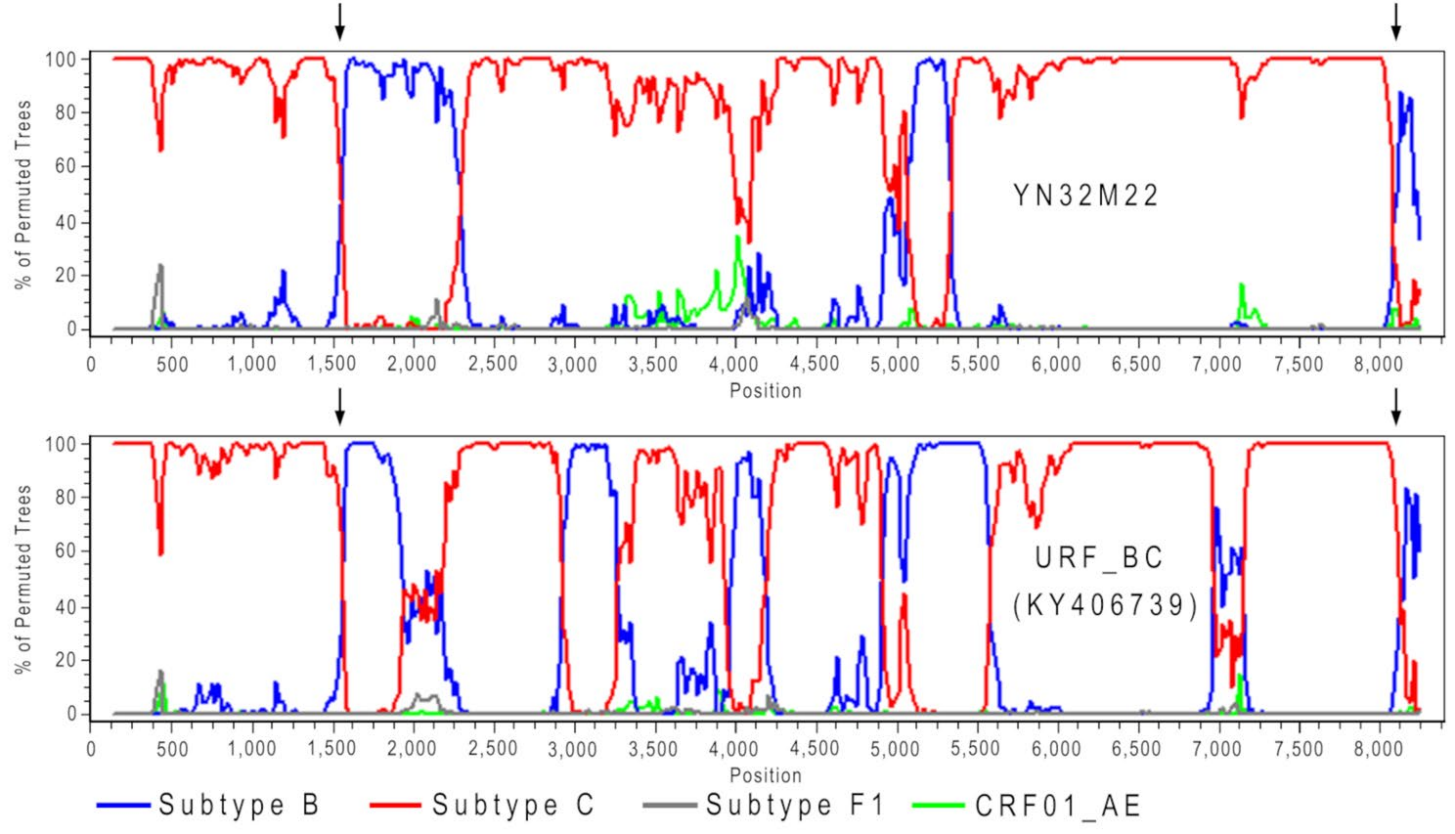
Bootscan plots of second-generation recombinant HIV sequences composed of URF_BC. In each plot, the lines in different colors represent the reference sequences of different subtypes, and the black arrows indicate the shared breakpoints among different HIV sequences

Bootscan analysis further supported this hypothesis. YN36F38 exhibited a recombination structure in the latter half of the genome identical to that of CRF82_cpx, with three shared recombination breakpoints (Fig. 3A). Similarly, YN35F22 shared a recombination structure in the latter half of the genome with CRF86_BC, with five shared recombination breakpoints (Fig. 3B). Meanwhile, YN34F21 and YN7F27 each shared four and three recombination breakpoints with CRF178_BC, respectively (Fig. 3C). YN32M22, on the other hand, shared two recombination breakpoints with KY406739 in both the anterior and posterior regions of the genome (Fig. 4). These findings provided further evidence that these five sequences (YN36F38, YN35F22, YN34F21, YN7F27, and YN32M22) represent recombinant strains resulting from second-generation recombination involving known CRFs or URFs.

Maximum likelihood trees constructed from the subregions of these five sequences revealed that their identical fragments clustered with their respective parental sequences (Figure S2). This observation further supports the conclusion that these sequences are second-generation recombinants derived from known CRFs or URFs.

### Newly formed URFs

The remaining sequence, YN8F28, did not cluster with any known subtypes, CRFs, or URFs. Instead, it positioned closer to the root of the phylogenetic tree, suggesting that it was genetically distant from known sequences and might have represented newly formed URF (Fig. 1).

**Fig. 5:**
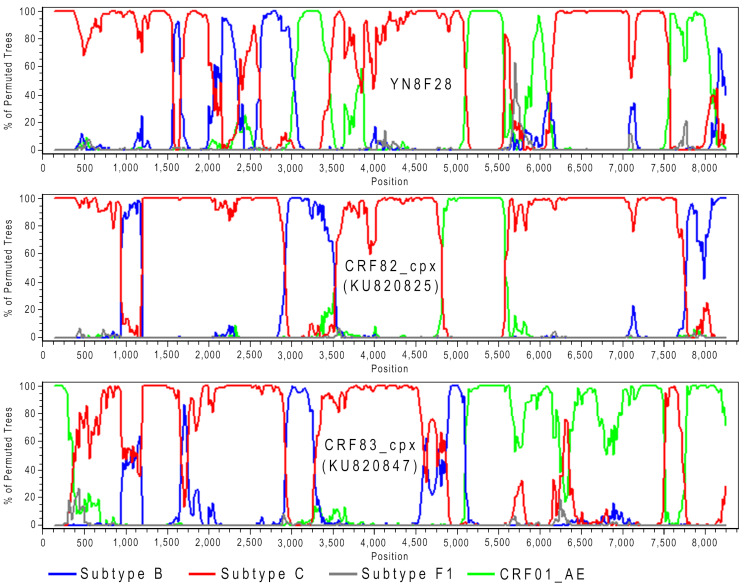
Bootscan plots of newly identified HIV URF and known CRFs. In each plot, the lines in different colors represent the reference sequences of different subtypes

Bootscan analysis confirmed that YN8F28 was a recombinant strain derived from HIV subtypes B and C, as well as CRF01_AE, comprising four subtype B fragments, six subtype C fragments, and four CRF01_AE fragments (Fig. 5). Although phylogenetically positioned between CRF82_cpx and CRF83_cpx (Fig. 1), YN8F28 shared no recombination breakpoints with these CRFs (Fig. 5). These findings indicated that YN8F28 was a novel URF arising from complex recombination events involving multiple HIV subtypes.

## Discussion

In 1989, the first indigenous cases of HIV in China were reported in Ruili, Yunnan, as an outbreak among 146 people who inject drugs, with subtype B being the predominant strain [14]. In 1992, subtype C began to spread among the same population in Ruili, and by 1994, a woman from Yunnan who had engaged in sex work in Thailand was confirmed to be infected with CRF01_AE [15, 16]. The co-circulation of subtypes B, C, and CRF01_AE in Yunnan led to the emergence of numerous recombinant strains, including CRF07_BC and CRF08_BC, which are currently among the major circulating strains in China [8, 17]. In this study, among eight Burmese individuals with HIV in Yunnan, we identified one subtype C and one CRF08_BC strain, while the other six were recombinant forms (Fig. 1). The patients infected with subtype C and CRF08_BC were diagnosed in 2006 and 2007, respectively, while the others were diagnosed in 2019 or 2020 (Table 1). Our findings align with previous studies, further confirming that recombinant strains have become the dominant HIV strains in Yunnan, China [6, 17–19].In 2017, we identified HIV CRF82_cpx and CRF83_cpx among people who use drugs in the Kokang Autonomous Region of Shan State, Myanmar, which borders Yunnan Province, China [9]. These newly named CRFs had become the predominant strains in the region at that time. Subsequently, in 2021, we discovered a recombinant strain involving CRF83_cpx among Burmese patients newly diagnosed with HIV in Dehong Prefecture, Yunnan Province [20]. In this study, we identified a second-generation recombinant strain involving CRF82_cpx among Burmese PLHIV in Baoshan, Yunnan Province (Fig. 3A). Furthermore, we also identified second-generation recombinant strains involving CRF86_BC and CRF178_BC (Fig. 3B and C). These results indicate that CRFs are not the endpoint of recombination but can serve as parental strains for new HIV recombinant strains.

Similarly, in 2017, Wang et al. identified a complex unique recombinant form (URF, KY406739) in an HIV-positive female in Yunnan, China [21]. In 2021, we identified a recombinant strain formed through second-generation recombination between URF (KY406739) and CRF86_BC in a patient newly diagnosed with AIDS in Baoshan, Yunnan [22]. In this study, we discovered a new URF involving KY406739 as well (Fig. 4), demonstrating that URFs are also not the endpoint of recombination but can serve as parental strains for new HIV recombinant strains.

Additionally, this study identified a URF with particularly complex recombination structures, each containing 13 recombination breakpoints (Figs. 5). Based on previous research and our own findings, we hypothesize that the primary reasons for the prevalence of complex HIV recombinant strains in the China-Myanmar border region may include the following: First, during the early stages of the HIV epidemic, multiple HIV subtypes (B, C, and CRF01_AE) were circulating in the region, potentially establishing a founder effect [16, 23]. Second, cross-border movements of different populations, such as drug users, long-distance truck drivers, and sex workers, further facilitated the “fusion” and recombination of different HIV subtypes [24–27]. Third, the CRFs and URFs formed through recombination serve as parental strains, contributing to the formation of new CRFs and URFs [7, 20, 22, 28, 29].

A key limitation of this study is the small sample size, which precludes temporal analysis of strain origins. Nevertheless, this study observed the ongoing formation of second-generation recombinants involving CRFs and URFs, as well as the emergence of complex URFs.

## Conclusion

A molecular epidemiological study of HIV was conducted among Burmese PLHIV in Baoshan, Yunnan Province, from 2006 to 2020. Results showed that recombinant strains were predominant, although subtype C and CRF08_BC were also circulating. Previously identified CRF82_cpx, CRF86_BC, and URF (KY406739) served as parental strains for new second-generation recombinant strains. These findings highlight the need for sustained molecular surveillance in the China-Myanmar border region to inform HIV prevention and control strategies.

## Electronic supplementary material

Below is the link to the electronic supplementary material.

Supplementary Material 1

Figure S1. Neighbor-joining tree constructed using near full-length HIV genomes. Red triangles indicate sequences amplified in this study; all other branches represent reference sequences, involving all known HIV subtypes and CRFs

Figure S2. Maximum-likelihood trees of subregions from the amplified sequences and their putative parental strains. Red triangles represent sequences amplified in this study. Genomic regions (relative to HXB2 coordinates) are indicated in parentheses

## Data Availability

The datasets generated and analyzed during the current study are available in the GenBank repository under accession numbers PQ757319-PQ757326. The datasets used and analysed during the current study are available from the corresponding authors on reasonable request.

## Abbreviations

AIDS: Acquired Immunodeficiency Syndrome
CRFs: Circulating Recombinant Forms
HIV: Human Immunodeficiency Virus
PLHIV: People Living with HIV
URFs: Unique Recombinant Forms
