# Supplementary material for: Genetic diversity of HIV in Yunnan, China: the role of second-generation recombination involving circulating and unique recombinant forms
Source: Virol J. 2025 Jul 14;22:240. doi: 10.1186/s12985-025-02863-y (PMC12257697; doi:10.1186/s12985-025-02863-y)
Supplement: Supplementary file 1 — Supplementary Material 1 [file 12985_2025_2863_MOESM1_ESM.docx]

**Table S1. Top five BLAST matches of the amplified sequences in this study, retrieved from the HIV sequence database.**

| Participant | Accession numbers of the amplified sequences | Accession numbers of the similar sequences |
| --- | --- | --- |
| YN7F27 | PQ757319 | KF250380, AY008716, AY008717, AY008715, FV536606 |
| YN8F28 | PQ757320 | MT624743, AY008717, AY008716, MT624746, KC899006 |
| YN9M24 | PQ757321 | AY008717, AY008716, AY008715, FV536606, AB773885 |
| YN32M22 | PQ757322 | KF250380, AY008716, AP005206, AY008717, AY008715 |
| YN33F28 | PQ757323 | KF250380, AF067157, KF835522, AB023804, AY713414 |
| YN34F21 | PQ757324 | PV207987, PV207988, AP005206, PV207989, AY008717 |
| YN35F22 | PQ757325 | KX582251, GU362013, KY406739, KU820848, AP005206 |
| YN36F38 | PQ757326 | KC898978, AB023804, AY008715, FV536606, AY008716 |
