# Supplementary figures and images for: Genetic diversity of HIV in Yunnan, China: the role of second-generation recombination involving circulating and unique recombinant forms
Source: Virol J. 2025 Jul 14;22:240. doi: 10.1186/s12985-025-02863-y (PMC12257697; doi:10.1186/s12985-025-02863-y)

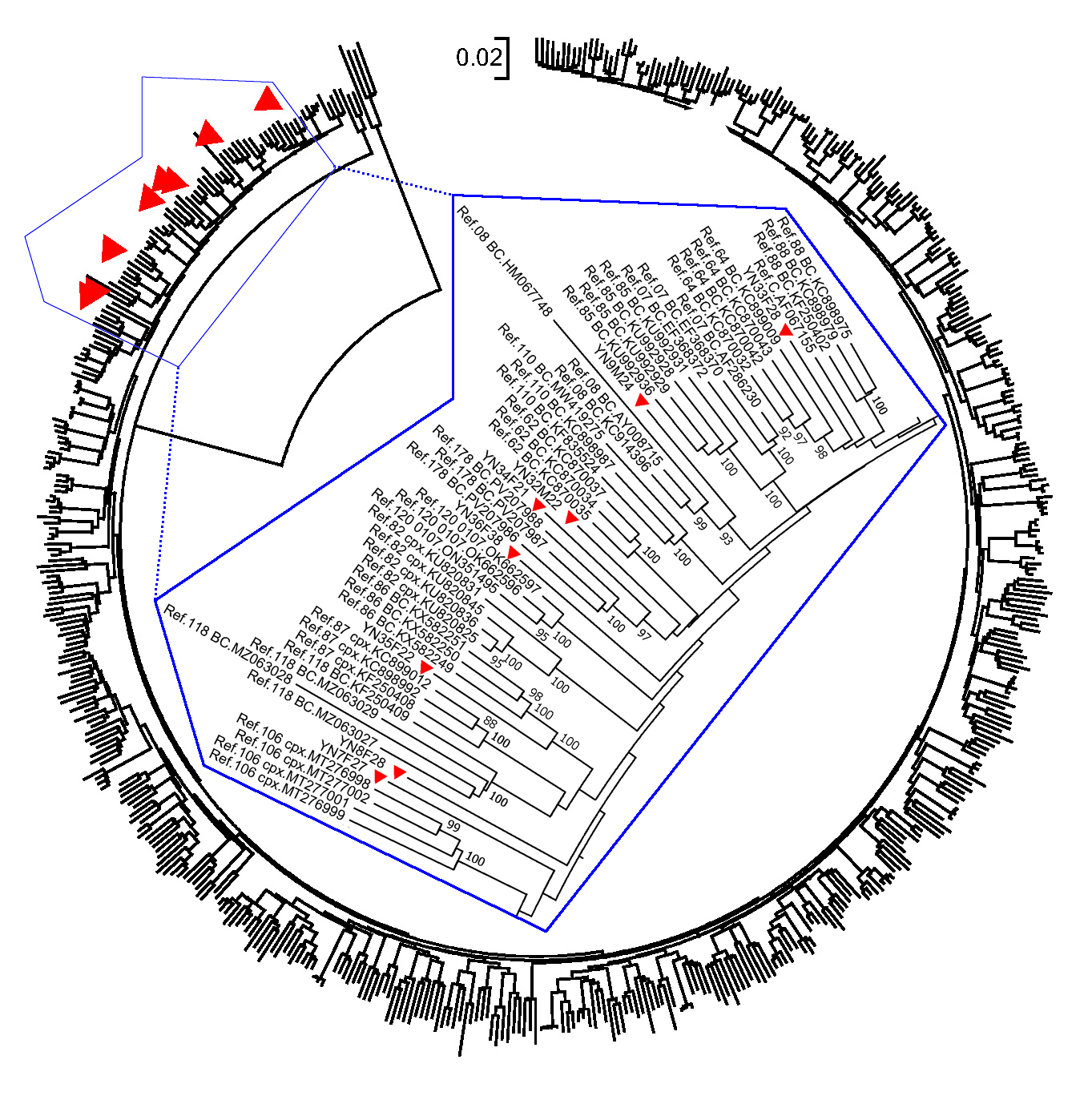

Supplement: Supplementary file 2 — Figure S1. Neighbor-joining tree constructed using near full-length HIV genomes. Red triangles indicate sequences amplified in this study; all other branches represent reference sequences, involving all known HIV subtypes and CRFs [file 12985_2025_2863_MOESM2_ESM.tif]

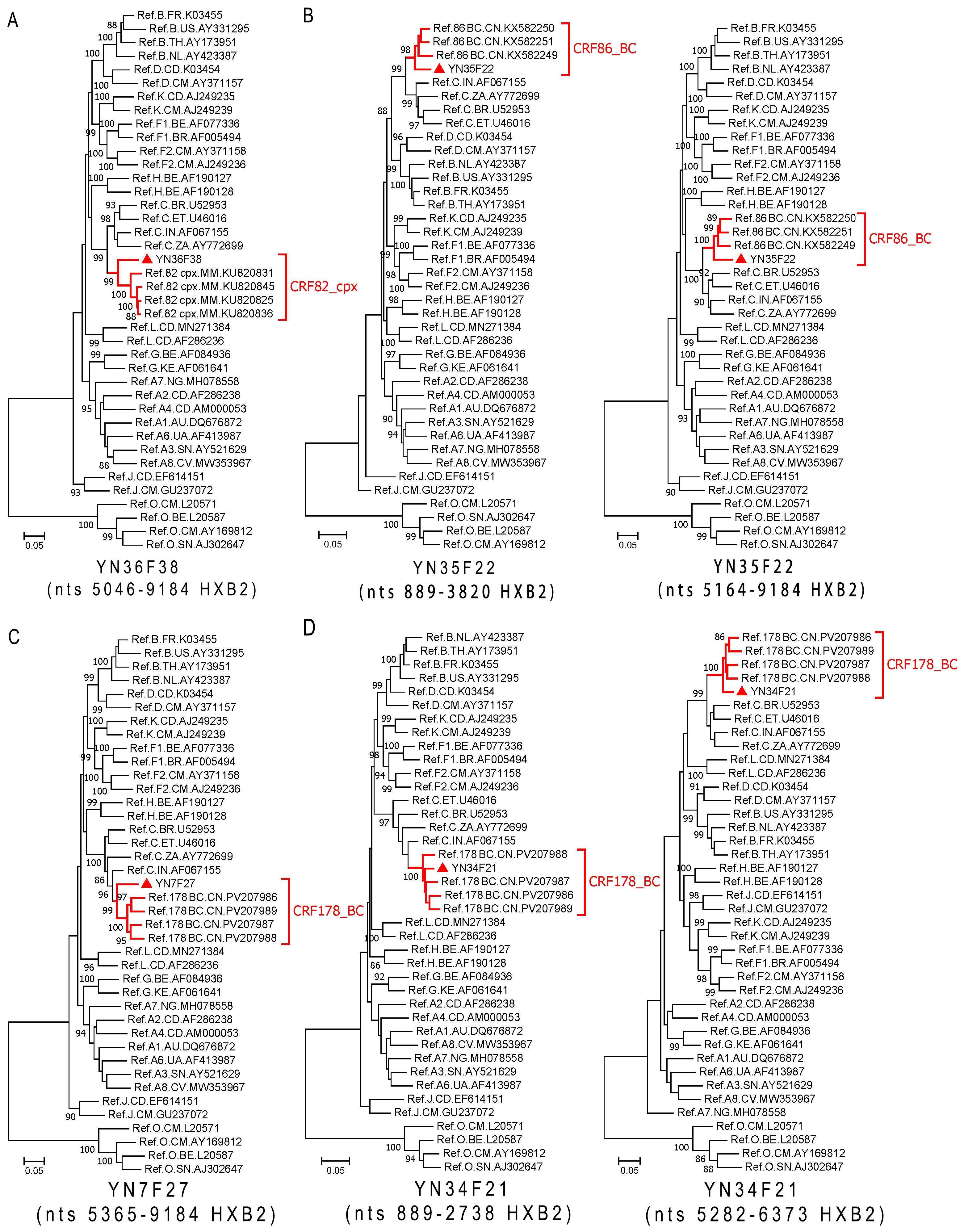

Supplement: Supplementary file 3 — Figure S2. Maximum-likelihood trees of subregions from the amplified sequences and their putative parental strains. Red triangles represent sequences amplified in this study. Genomic regions (relative to HXB2 coordinates) are indicated in parentheses [file 12985_2025_2863_MOESM3_ESM.tif]
